# Insecticidal efficacy of afoxolaner against bedbugs, *Cimex lectularius*, when administered orally to dogs

**DOI:** 10.1051/parasite/2021004

**Published:** 2021-02-02

**Authors:** Frederic Beugnet, Carin Rautenbach, Luther van der Mescht, Wilfried Lebon, Nesrine Aouiche, Julian Liebenberg

**Affiliations:** 1 Boehringer-Ingelheim 29 Av. Tony Garnier 69007 Lyon France; 2 ClinVet PO Box 11186 Universitas 9321 Bloemfontein South Africa

**Keywords:** Dogs, Bedbugs, *Cimex lectularius*, Afoxolaner, Insecticidal activity

## Abstract

The objective of this experimental study was to assess the insecticidal efficacy of afoxolaner (NexGard^®^) against bedbugs (*Cimex lectularius*) on dogs. For each challenge, 20 bedbugs were placed in two chambers positioned in contact to the dog’s skin for 15 min, after which live fed parasites were counted and incubated for survival evaluations. On Day 0, 7 dogs assigned to the treated group were administered afoxolaner orally at the registered dose. All 14 dogs were challenged on Days 1, 7, 14, 21 and 28, and the collected live fed *C. lectularius* incubated for 72 h (Day 1), and 72 h and 96 h (Days 7, 14, 21 and 28) for survival evaluation. The percent feeding in the control group ranged from 95% to 98.6% and the percent of live fed bedbugs at 96 h ranged from 99.3% to 100% in the control group, demonstrating the viability of the strain and their capacity to feed on dogs. Significantly fewer live fed bedbugs were counted in the treated group, compared to the control group, at all time-points. The reduction of live fed *C. lectularius* in the afoxolaner group was 41.4% at 72 h after the Day 1 challenge, and 77.2%, 82.7%, 85.0% and 63.5% at 96 h after the Days 7, 14, 21 and 28 challenges, respectively. It is hypothesized that monthly treatment of dogs with afoxolaner could help in preventing a bed bug population from installing in a household if bedbugs bite dogs in the presence of humans.

## Abstract (fr)

L’objectif de cette étude expérimentale était de déterminer l’efficacité insecticide de l’afoxolaner (NexGard^®^) contre les punaises de lit (*Cimex lectularius*) chez les chiens. Pour chaque exposition*,* 20 punaises de lit ont été mises dans deux chambres placées en contact avec la peau des chiens pendant 15 minutes. Après cela, les parasites vivants et gorgés ont été comptés et incubés pour évaluer leur survie. Le jour 0, 7 chiens affectés au groupe traité ont reçu de l’afoxolaner (NexGard) par voie orale à la dose commerciale. Les 14 chiens ont été exposés aux punaises aux jours 1, 7, 14, 21 et 28, et les *C. lectularius* vivants et gorgés, collectés, ont été incubés pendant 72 h (jour 1) et 72 et 96 h (jours 7, 14, 21 et 28) pour l’évaluation de la survie. Le pourcentage d’engorgement dans le groupe témoin variait de 95 % à 98,6 % et le pourcentage de ces punaises vivantes à 96 h variait de 99,3 à 100 %, démontrant la viabilité de la souche et la capacité à se nourrir des chiens. Le nombre de punaises vivantes était significativement plus faible dans le groupe traité, par rapport au groupe témoin, à chaque point de contrôle. La réduction de *C. lectularius* vivants dans le groupe afoxolaner était de 41,4 % à 72 h après l’exposition du jour 1, et respectivement de 77,2 %, 82,7 %, 85,0 %, et 63,5 % à 96 h après les expositions des jours 7, 14, 21, et 28. On peut donc faire l’hypothèse que le traitement mensuel des chiens avec de l’afoxolaner pourrait empêcher une population de punaises de lit de s’installer dans un foyer, si les punaises piquent les chiens en présence d’humains.

## Introduction

Bedbugs are hematophagous arthropods. The discovery of specimens in tombs at Tell al-Armana, Egypt, suggests that these insects have been pestering humans for at least 3550 years [1, 14]. With social progress and insecticide development, bedbugs became uncommon in developed countries in the 1950s. Nevertheless, a clear bed bug resurgence has been observed since 2000 in many western countries [1, 12].

Bedbugs, *Cimex lectularius* Linnaeus, 1758 and *Cimex hemipterus* Johan Christian Fabricius, 1803*,* belong to the Hemiptera order of insects, in the Cimicidae family. They are related to Reduviidae, i.e. kissing bugs like *Triatoma* spp. and *Rhodnius* spp., the vectors of Chagas disease (*Trypanosoma cruzi* infection) in Central and South America. Unlike in the case of kissing bugs, no vector role is known for bedbugs [7]. They do, however, represent a major nuisance due to their bites inducing papules, erythema, pruritus, pain, but also psychologic fear [1, 20]. Typically, the bites frequently follow a line or curve [20].

Adult *C. lectularius* and *C. hemipterus* are reddish-brown, flat, wingless, oval insects (4–7 mm). Both males and females are hematophagous and can live for 12 months without feeding and even 1.5–2 years in colder environments. Under a constant temperature of 14–27 °C, eggs hatch 4–10 days after mating, yielding the nymphs, which are 1–3 mm long, and lighter than adults in color. Each of the 5 nymphal stages require a blood meal, which can last 10–20 min, to be able to moult to the next stage in about 3–7 days. Bedbugs fear light and are generally active in the dark [1, 14]. They hide in any small dark place, such as bedclothes, mattresses, springs, bed frames, cracks, crevices, sofa, carpets, and wallpaper. Bedbugs can travel long distances when being dispersed by human clothes, luggage, or furniture. Overcrowding and derelict living conditions may be factors that increase the bed bug burden in an area [1, 13].

Bed bug eradication from a contaminated site is a challenge due to their ability to hide and the difficulty to kill all insects in the environment. Insecticide resistance is also an increasing problem [6, 8]. Successful bed bug elimination relies mainly on good cooperation between the owner of the contaminated site and the pest manager for site assessment, thorough inspection, identification, and eradication [13]. In heavily contaminated environments, an “efficient search-and-destroy” operation must be imposed, starting by removing all bed linens and washing them at a temperature higher than 60 °C. Checking and dismantling all furniture is the next step to access all bedbug hiding places, to identify and destroy eggs, nymphs, and adults. It is always best to vacuum the affected area first to reduce the overall bed bug population. The use of persistent insecticides provides residual protection against survivors, but integrated pest management is key and we cannot only rely on the use of pesticides. In buildings, it may be advisable to treat adjoining apartments or rooms, even when no bedbugs were found during the inspection [1].

Bedbugs probably originated as ectoparasites of bats, infesting humans when they co-habitated in caves [1, 14]. As humans quickly domesticated dogs and cats in the prehistoric period, *Cimex* insects may also have been in contact with these mammals in ancient times. They thus may opportunistically feed on household pets like dogs and cats, which needs to be better assessed in the field [1, 14]. Therefore, it may be interesting to study the effectiveness of systemic insecticides like NexGard administered to dogs against bedbugs. The impact on bed bug populations would need to demonstrate that bedbugs bite dogs in the presence of humans who are considered to be the preferred hosts.

Isoxazolines represent the most recent chemical group of insecticides developed for use in domestic animals. Their mode of action is systemic, after oral administration, or through transcutaneous absorption [22]. To date, the only assessment of the insecticidal activity of a systemic insecticide against bedbugs was conducted using an *in vitro* design with a macrocyclic lactone, moxidectin [23]. No assessment has been conducted under real conditions with an *in vivo* design. Isoxazolines are highly bound to plasma proteins (i.e. 99%), have a long half-life (i.e. 9–14 days for oral afoxolaner), and provide high insecticidal activity against hematophagous parasites [16, 22]. Afoxolaner, formulated as a palatable chewable tablet kills existing and new infesting fleas and ticks for a month [10]. Additionally, it is effective against mites (i.e. *Sarcoptes scabiei*, *Demodex canis*, *Otodectes cynotis*) [2, 3, 5], and has demonstrated insecticidal activity against hematophagous flying insects (i.e. sandflies, *Phlebotomus perniciosus*, and mosquitoes, *Aedes aegypti*) through the treatment of dogs [17, 21]. Recently, an insecticidal effect of afoxolaner and fluralaner has also been demonstrated in treated dogs against kissing bugs (*Triatoma infestans*), the vector of Chagas disease [15, 18]. We therefore hypothesized that the blood of isoxazoline-treated dogs could be able to kill bedbugs. This study was conducted to determine the insecticidal efficacy of NexGard^®^ (Boehringer Ingelheim Animal Health) against bedbugs (*C. lectularius*) feeding on treated dogs.

## Materials and methods

The study was approved by the Clinvet Institutional Animal Care and Use Committe (IACUC), and followed European Union Directive 2010/63/EU on the protection of animals used for scientific purposes [11]. It was conducted in accordance with the VICH GL9 guideline on Good Clinical Practices (https://www.ema.europa.eu/en/vich-gl9-good-clinical-practices).

The study was a blinded, randomized, negative-controlled, efficacy study. The design was inspired by the European Medicine Agency (EMA) and the World Association for the Advancement of Veterinary Parasitology (WAAVP) guidelines for the testing and evaluation of the efficacy of antiparasitic substances against ectoparasites [9, 19]. The study included two groups of seven dogs each, selected from an initial group of 16 dogs. Group 1 dogs were sham-dosed (negative control), whereas group 2 dogs were treated orally once with afoxolaner (NexGard^®^, Boehringer Ingelheim Animal Health) at label dose. Sixteen dogs (males and females) were acclimatized to their cages from Day −7. On Day −6, an initial exposure to 20 *C. lectularius* was conducted to evaluate the susceptibility of each dog to experimental infestation, for random allocation of the dogs to the study groups, and to test the model. The two dogs with the lowest number of live fed bedbugs at 15 min post-exposure were removed.

Fourteen healthy dogs, 5 beagles and 9 mixed-breeds, 7 males and 7 females, weighing between 11.90 kg and 17.65 kg, >6 months, that had not been treated with a topical or systemic acaricide/insecticide over the 12 weeks preceding Day 0 were randomly allocated to groups 1 and 2 on the basis of *C. lectularius* live fed pre-treatment counts.

On Day 0, all dogs assigned to group 2 were administered a 68 mg NexGard chew, in order to deliver at least 2.5 mg/kg afoxolaner, in accordance with the label [10]. Dogs in group 1 were sham-dosed by bringing the animal to the table, opening the mouth and then returning the animal to its cage. Dogs were observed hourly for 4 h after NexGard/sham-dose administration, and daily from Day −7 to Day 28 for general health and adverse reactions.

**Figure 1:**
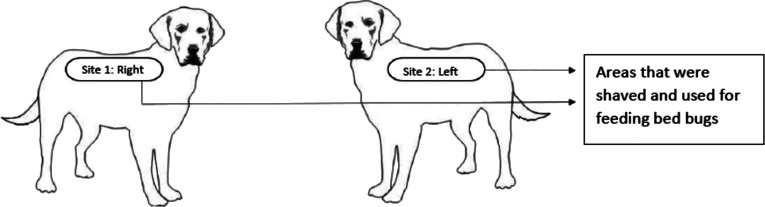
Areas of feeding of the bedbugs on dogs.

Dogs were exposed to 20 unfed, adult *C. lectularius* on Days 1, 7, 14, 21 and 28 to assess insecticidal activity. The bedbugs were fed on rabbits one week earlier, following the breeding process of the colony, including one blood meal per week. For each *C. lectularius* challenge, 20 unfed adult bedbugs were placed in chambers positioned in close contact to the dog’s skin for at least 15 min, after which time live fed parasites were counted and incubated for survival evaluation. Feeding assessment on all bedbugs was performed immediately post-challenge by visual observation of the fed state of the bedbugs. The live fed bedbugs were then incubated in an insectarium and viability assessments were performed at 72 h (Day 1), and 72 h and 96 h (Days 7, 14, 21, 28) post-feeding. For each challenge, dogs were sedated with medetomidine (Domitor^®^, 100 μg/kg, 0.1 mL/kg intramuscular injection prior to exposure to the bedbugs, at the end of which, atipamezole (Antisedan^®^, 200 μg/kg, 0.04 mL/kg intramuscular injection) was used to reverse the sedation. The sedated dogs were placed in a dark room to facilitate feeding of the bedbugs. Two chambers, covered with an appropriate material (mesh netting) through which the bedbugs could feed, containing ten adults (5 males and 5 females) each, were held against the shaved areas of the dog, by applying light pressure to ensure sufficient contact with the dog’s skin. The shaving is related to the mesh netting of the chambers which would flatten the hairs and form a barrier on unshaved dogs/rabbits. After each feeding phase, the dog’s skin was examined for any abnormality. No adverse reactions to the feeding of the bedbugs were observed. Shaved areas for placement (i.e. Site 1 [right side of dog] and Site 2 [left side of dog]) of feeding chambers on each dog, were prepared, on Day −7, and then re-used for all subsequent exposures (Fig. 1).

The *C. lectularius* strain had originally been collected in human habitats in Bloemfontein, South Africa in 2017, and since then was maintained in an insectarium and fed weekly on rabbits using classical methods to breed bedbugs [4]. Adult unfed male and female bedbugs were used in the challenges.

The fed and live bedbugs were incubated in an insectarium with a controlled temperature (22.0 °C to 26.9 °C) and humidity (67.3–76.2% relative humidity) and assessed for live/dead status at 72 h and 96 h [4].

Following the WAAVP guideline on how the assess insecticidal efficacy, the primary efficacy criterion was comparison of the number of live fed bedbugs counted in the treated group compared to the negative control on the various assessment days [9, 19]. The difference in dead bed bug counts was considered a secondary criterion.

The insecticidal efficacy against bedbugs was calculated at each assessment day according to the formula given below. Efficacy calculations were based on arithmetic mean values:

$$\mathrm{Insecticidal}\;\mathrm{efficacy}\;(\%)\;\mathrm{against}\;\mathrm{bedbugs}=100\times (M_{c}-M_{t})/M_{c},$$

where:

*M*_*c*_ = mean number of live, fed bedbugs in the control group at each assessment time point (72 h and 96 h after the 15 min feeding phase);
*M*_*t*_ = mean number of live, fed bedbugs in the treated group at each assessment time point (72 h and 96 h after the 15 min feeding phase).

Due to the lack of bed bug specific guidelines, the Committee for Medicinal Products for Veterinary Use (CVMP) guideline “Guideline for the testing and evaluation of the efficacy of anti-parasitic substances for the treatment and prevention of tick and flea infestation in dogs and cats” suggestion that at least six animals were to be used per group was followed.

The groups were compared using an ANOVA with a treatment effect on logarithmic transformed fed bed bug (count + 1) data. The level of significance of the formal tests was set at 5%. All tests were two-sided.

## Results

No significant difference was recorded between the two groups regarding dog body weights (*p* = 0.7128) and live fed bed bug counts (*p* = 0.8597) during the assessment performed in the acclimation period. This indicated homogenous distribution between the two groups.

No abnormal signs were observed during the daily observations, during the specific post-administration observations. No adverse skin reactions were observed on the bedbugs feeding site.

### Bed bug challenges and viability assessments

**Figure 2:**
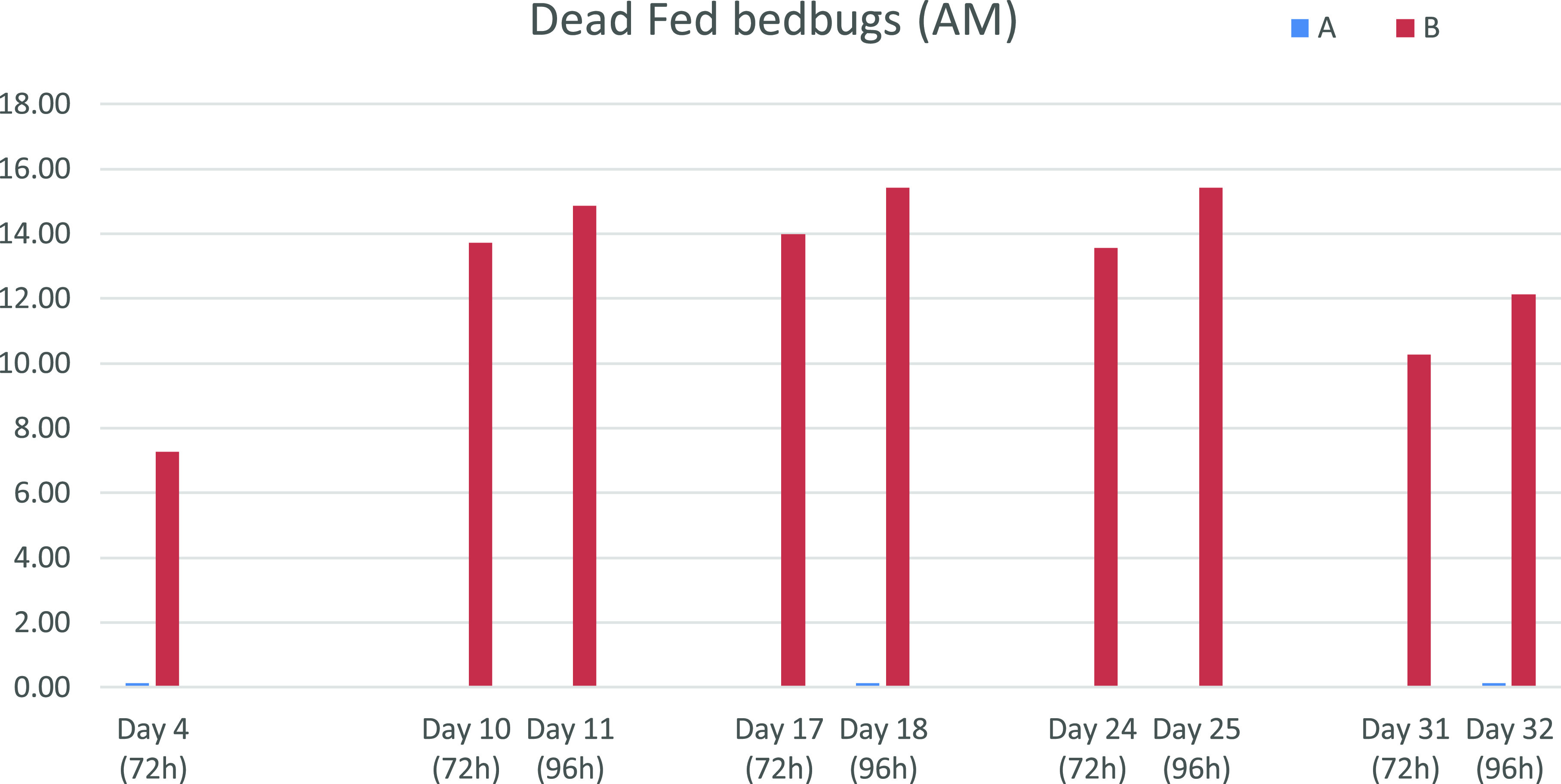
Number of dead fed bedbugs counted in the control group (A-blue) and the treated group (B-red).

Percentage of feeding in control group 1 ranged from 95.0% to 98.6% at all assessment time points, demonstrating good host tropism for dogs (Supplementary Figs. 1 and 2). Percentage of live bedbugs observed at 72 h and 96 h in control group 1 ranged from 99.3% to 100%, demonstrating the very good viability of the bedbugs (Table 1, Fig. 2).

**Table 1:** % Insecticidal and % mortality observed on bedbugs at 72 h and 96 h post 15 min feeding exposure to dogs (arithmetic means).

	Day 1 + 72 h	Day 7 + 72 h	Day 7 + 96 h	Day 14 + 72 h	Day 14 + 96 h	Day 21 + 72 h	Day 21 + 96 h	Day 28 + 72 h	Day 28 + 96 h
Control									
Mean number Live fed	19.00	19.40	19.40	19.10	19.00	19.00	19.00	19.70	19.60
Mean number Dead fed	0.10	0.00	0.00	0.00	0.10	0.00	0.00	0.00	0.10
Mean number Unfed	0.90	0.50	0.50	0.80	0.80	1.00	1.00	0.30	0.30
Treated									
Mean number Live fed	11.10	5.60	4.40	4.70	3.30	4.70	2.90	9.00	7.10
Mean number Dead fed	7.30	13.70	14.90	14.00	15.40	13.60	15.40	10.30	12.10
Mean number Unfed	1.50	0.70	0.70	1.30	1.30	1.70	1.70	0.70	0.70
% Insecticidal efficacy	**41.4**	71.13	**77.2**	75.4	**82.7**	75.2	**85.0**	54.3	**63.5**
*p*-value	0.0059	0.0059	0.0059	0.0059	0.0059	0.0059	0.0059	0.0059	0.0059
% Mortality treated fed	**39.7**	71.0	**77.2**	75.0	**82.3**	74.3	**84.1**	53.4	**63.0**
*p*-value	0.001	<0.0001	<0.0001	<0.0001	<0.0001	<0.0001	<0.0001	<0.0001	<0.0001

*p*-value: One-way ANOVA with a treatment effect.

Mean numbers in arithmetic means.

Bold values indicate that it is the last time-point after each exposure day.

A significantly smaller number of live fed bedbugs was recorded for treated group 2, compared to negative control group 1 at all assessment time points (*p* ≤ 0.02) and significantly more dead fed bugs were counted in the treated group (Table 1).

**Figure 3:**
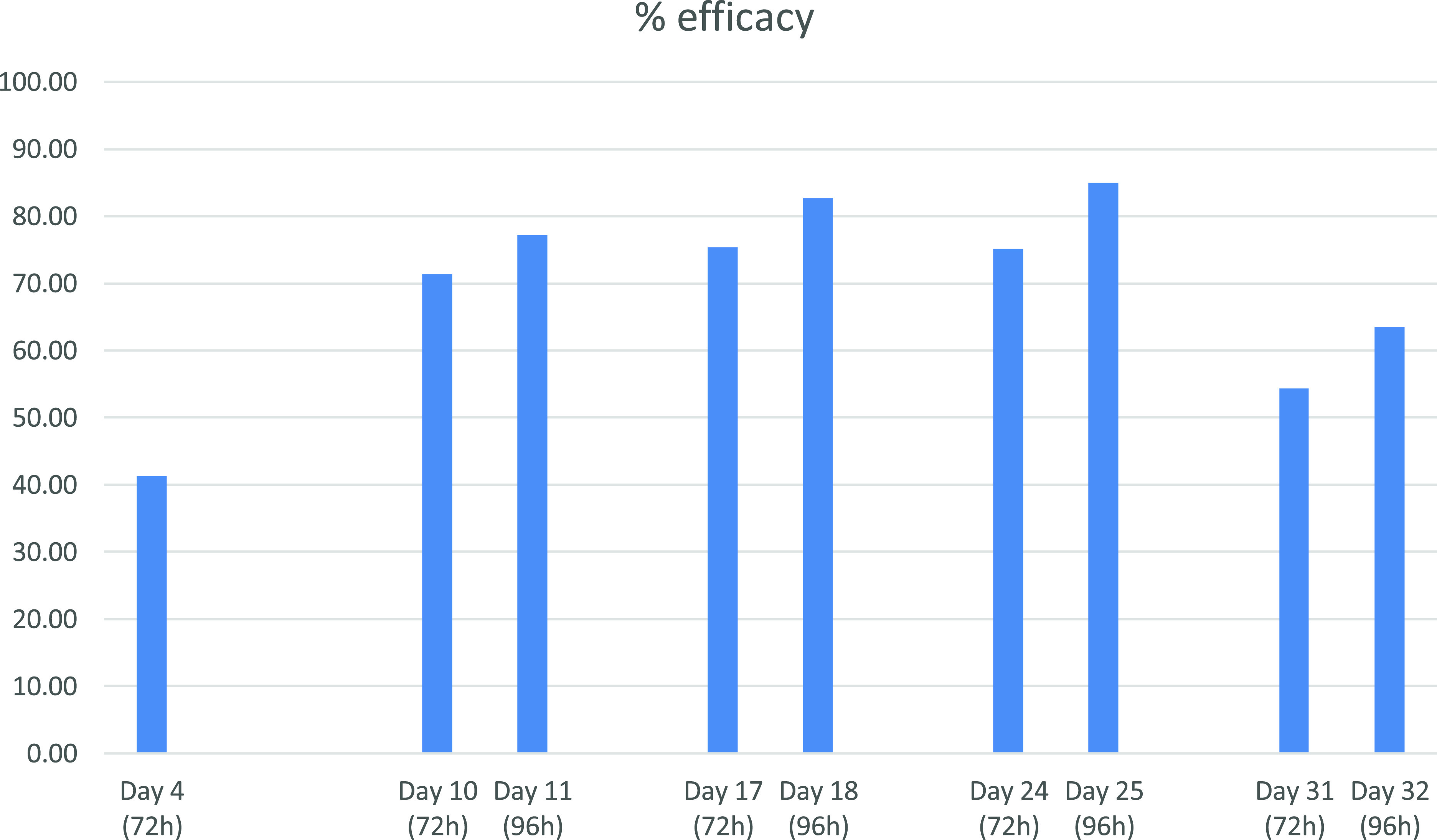
% NexGard efficacy against bedbug feeding on treated dogs.

The insecticidal efficacy observed on Day 1 was 41.4% at 72 h after the feeding phase (no evaluation was performed at 96 h). On the subsequent infestations, afoxolaner showed efficacy ranging between 63.5% and 85.0% at 96 h (Fig. 3).

## Discussion

There are limited data on the infestation of dogs and cats by *C. lectularius* bedbugs. They are thought to bite pets, but the frequency remains unknown [1, 17]. The fact that bedbugs are maintained in the laboratory using weekly feeding on rabbits provides initial evidence that mammals other than humans may be functional hosts [4]. In this study, 18–20 of the bugs (>95% in all challenges) did take a blood meal in both groups at each challenge, demonstrating a high tropism for dogs, with no natural repellent effect that could have been related to skin thickness or dog odour. Dogs were shaved smoothly, avoiding any skin damage or irritation, on a small surface corresponding to the vials containing the bedbugs, exactly as performed on rabbits. The shaving was performed to ease the application of the vial, avoiding bedbug escape. We do not consider that bedbugs would have any difficulty moving past hair, exactly as ticks do. Morphologically bedbugs are dorsoventrally flat and in that sense close to the form of ticks. Under natural conditions, they may also have easy access to body parts that are not hairy like the abdomen, especially when the dogs sleep.

There was no bedbug feeding rate difference observed between the two groups. Afoxolaner is an insecticide acting through blood ingestion, therefore no repellent effect is expected against insects [16, 22]. The absence of a difference in feeding rates between control dogs and treated dogs had already been observed in insecticidal efficacy studies against mosquitoes and sandflies [17, 21].

Feeding on untreated dogs did not impair the viability of the bedbugs. The arithmetic mean of dead bedbugs in the untreated control group ranged between 0 and 0.1 at 96 h, corresponding to a maximum of 1 dead bed bug for 120 bedbugs used in each exposure (20 × 6 dogs).

Afoxolaner demonstrated significant insecticidal activity at all time-points. The lowest efficacy was observed after the Day 1 challenge, which may be related to the pharmacokinetic properties of the treatment and a lower plasma concentration of the active substance. Thereafter, afoxolaner efficacy was above 77.2% at 96 h for 3 weeks, with a peak at 85.0%, decreasing to 63.5% at the end of the month. The insecticidal activity was greater at 96 h than 72 h, indicating gradual death of the bedbugs. These observations are consistent with the known significant correlation between afoxolaner plasma concentrations and efficacy [16], and was already observed in the efficacy studies against mosquitoes and sandflies [17, 21]. This is not the case in flea and tick efficacy studies as the minimum dose of afoxolaner (i.e. 2.5 mg/kg) was determined to provide sustained efficacy >95% against fleas within 24 h and >90% against ticks within 48 h for a full month [10, 16, 22]. The actual mortality percentages could be higher than those observed at 96 h, if the bugs had been incubated for longer observations.

As bedbugs may take a blood meal each week or more frequently [1, 4, 20], it would have been interesting to study the efficacy after a second bite on the bedbugs that had survived the first one, but this was not originally planned in this study design.

Our study results are the first demonstration of insecticidal efficacy of an isoxazoline compound, afoxolaner, against bedbugs. This opens the door for new possibilities to control bed bug infestations by regularly treating dogs living in households. Nevertheless, further assessments need to be carried out, especially to verify that bedbugs take blood meals on dogs in the presence of humans. This could be performed through PCR techniques on collected bedbugs, identifying specific dog markers. The authors are working on this type of epidemiological survey and are collecting bedbugs in apartments and households where dogs and cats are present. Another next step could be to assess the capability of bedbugs to take a blood meal on cats, as they do on dogs, and to assess the efficacy of isoxazoline against bedbugs through treated cats. Finally, an additional important step will be to perform investigations to measure the effect of this potential new mode of control under real situations through epidemiological surveys.

## Supplementary Material

Supplementary material is available at https://www.parasite-journal.org/10.1051/parasite/2021004/olm *Supplementary Figures 1 and 2*. Experimental device with adult bedbug feeding on dogs.

## Competing interest

The work reported herein was funded by Boehringer Ingelheim Animal Health. Wilfried Lebon, Nesrine Aouiche and Frédéric Beugnet are current employees of Boehringer Ingelheim. The other authors are employees of the Contract Research Organization Clinvet. NEXGARD^®^ is a registered trademark of Boehringer Ingelheim. All other trademarks are the property of their respective owners. This document is provided for scientific purposes only. Any reference to a brand or trademark herein is for informational purposes only and is not intended for a commercial purpose or to dilute the rights of the respective owner(s) of the brand(s) and trademark(s).
