# Supplementary figures and images for: Insecticidal efficacy of afoxolaner against bedbugs, Cimex lectularius, when administered orally to dogs
Source: Parasite. 2021 Feb 2;28:7. doi: 10.1051/parasite/2021004 (PMC7852378; doi:10.1051/parasite/2021004)

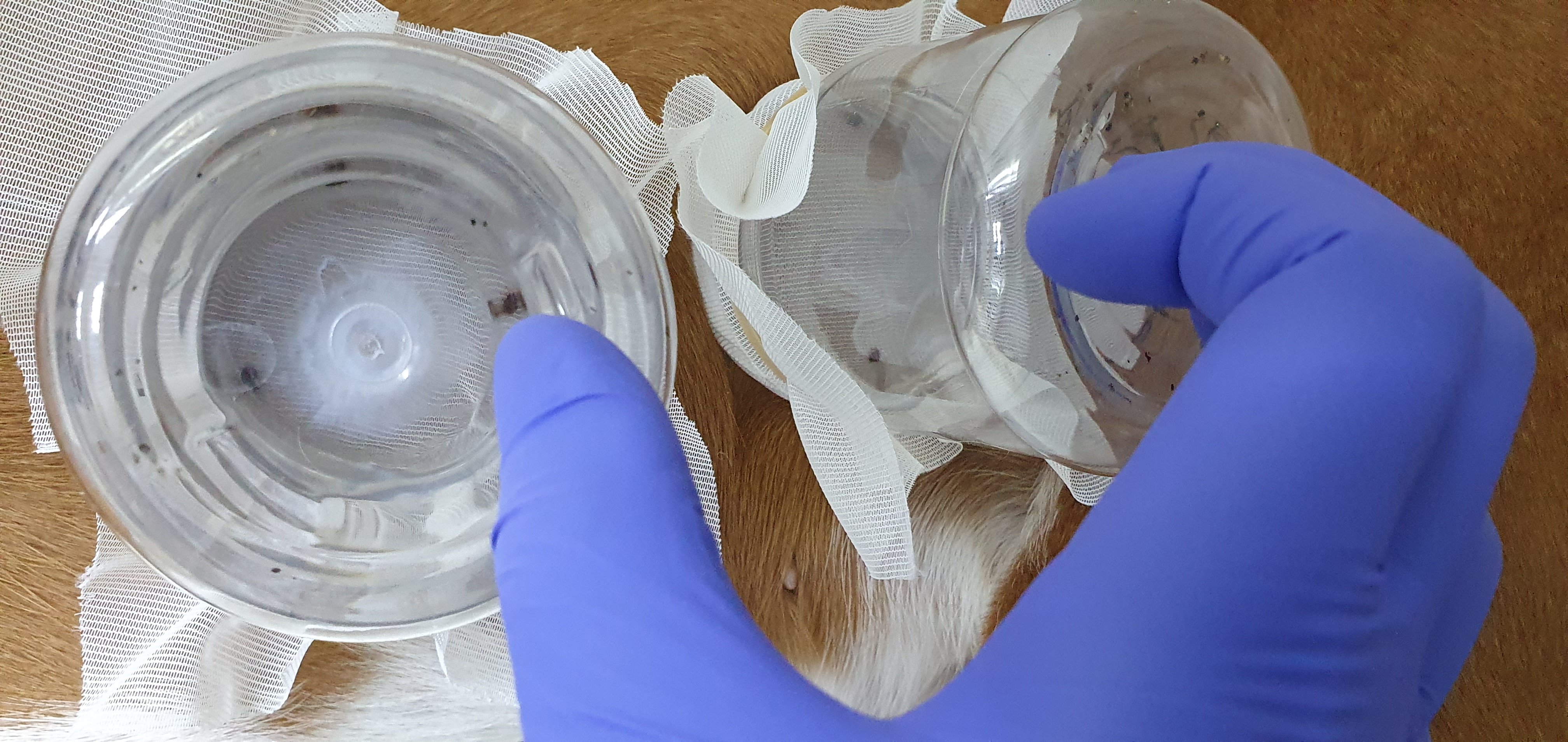

Supplement: Supplementary file 1 — Supplementary Figures 1 and 2. Experimental device with adult bedbug feeding on dogs. [file parasite-28-7-s1.zip › parasite200176-1-olm/Photo 1 - Experimentalfeeding on dog.jpg]

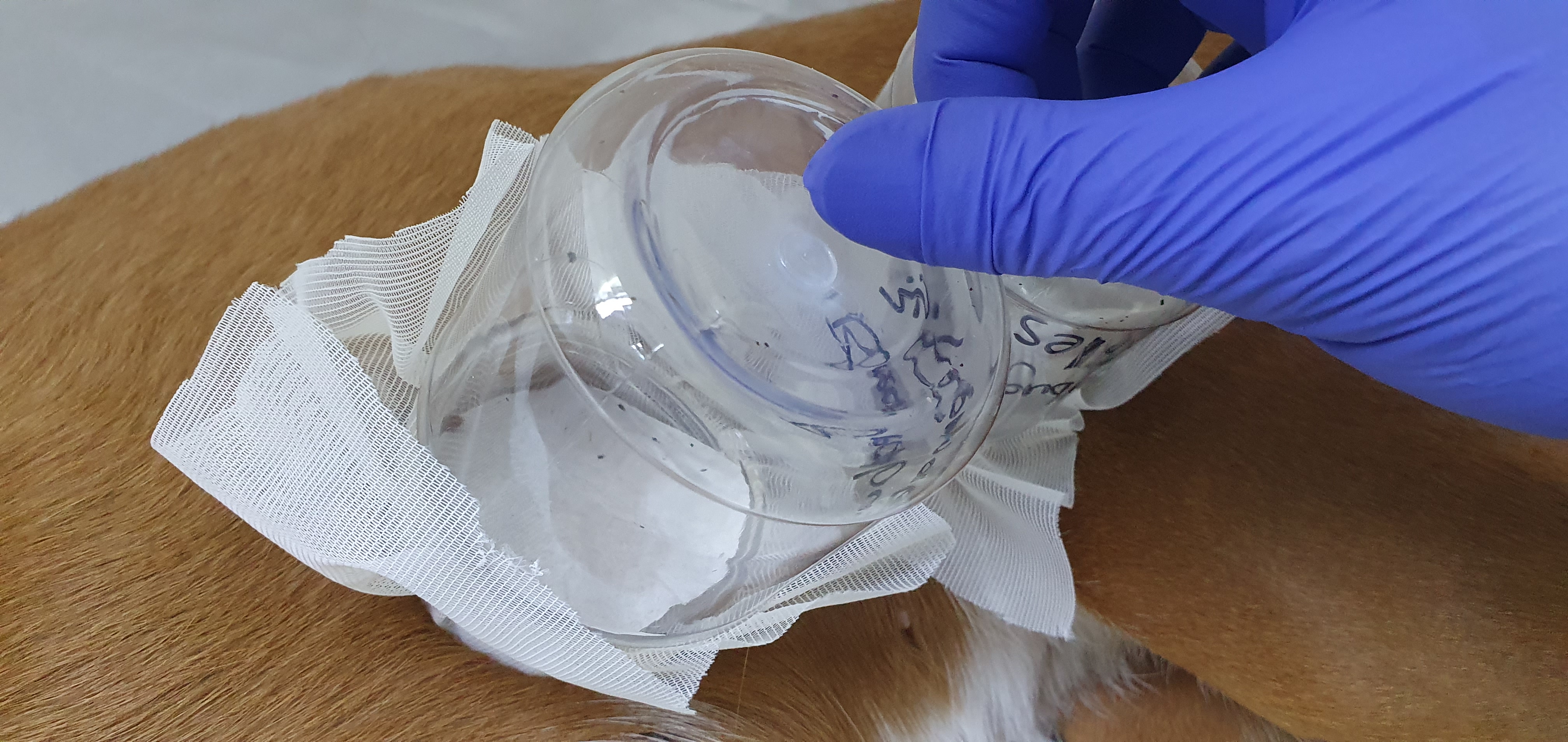

Supplement: Supplementary file 1 — Supplementary Figures 1 and 2. Experimental device with adult bedbug feeding on dogs. [file parasite-28-7-s1.zip › parasite200176-1-olm/Photo 2 - Experimental feeding on dog.jpg]
